# Cavernous Sinus Thrombosis Following the Use of Oral Contraceptive Pills in a Young Woman: A Case Report

**DOI:** 10.1002/ccr3.73478

**Published:** 2026-09-09

**Authors:** Zohreh Akbari, Ali Salami, Ali Movahedi, Seyyedeh Maryam Seyyedy, Kiana Babaei

**Affiliations:** ^1^ Department of Anesthesia Neyshabur University of Medical Sciences Neyshabur Iran; ^2^ Department of Neurosurgery, Imam Ali Hospital North Khorasan University of Medical Sciences Bojnurd Iran; ^3^ Department of Operating Room Neyshabur University of Medical Sciences Neyshabur Iran

**Keywords:** cavernous sinus thrombosis, cerebral venous sinus thrombosis, headache, intracerebral hemorrhage, oral contraceptive pills

## Abstract

Cavernous sinus thrombosis (CST) is a rare and potentially life‐threatening subtype of cerebral venous sinus thrombosis that often poses diagnostic challenges in emergency settings. We report a 34‐year‐old woman with a one‐year history of oral contraceptive pill (OCP) use for polycystic ovary syndrome who presented with severe headache, seizures, decreased level of consciousness, and bilateral eyelid edema. Non‐contrast brain computed tomography showed extensive intracerebral hemorrhage with findings suggestive of CST. Because of clinical instability, advanced venous imaging (CTV/MRV) could not be performed, and the diagnosis remained presumptive. The patient was treated with intravenous unfractionated heparin, intracranial pressure control and subsequent decompressive craniectomy because of progressive neurological deterioration. This case highlights the need for a high index of suspicion, prompt imaging, and early anticoagulation in patients with suspected CST and prothrombotic risk factors such as OCP use.

## Key Clinical Message

In women receiving oral contraceptive pills, cavernous sinus thrombosis should be suspected when severe headache, seizures, altered consciousness, or periorbital edema develops, particularly during periods of dehydration or fasting. Prompt neuroimaging, early anticoagulation even in the presence of intracerebral hemorrhage, and timely neurosurgical intervention may be life‐saving.

## 1 Introduction

Cerebral venous sinus thrombosis (CVST) is an uncommon but increasingly recognized cerebrovascular disorder, with incidence estimates ranging from 3 to 4 [1] to 13 or more cases per million population [2, 3], and a reported prevalence of 12.3 per million in Iran [4]. Cavernous sinus thrombosis (CST) is a rare and potentially life‐threatening subtype, accounting for only 1%–4% of CVST cases [5], that may arise from septic causes (e.g., sinusitis, facial infection) or aseptic causes such as thrombophilia, pregnancy, and hormonal contraceptive use [6]. Oral contraceptive pills (OCPs), used by an estimated 151 million women worldwide [7] and commonly prescribed for polycystic ovary syndrome and other gynecological conditions [8], are a well‐established risk factor for venous thrombosis observational data show they increase the risk of CVST by up to 5‐ to 22‐fold [9]. Because CST can present with nonspecific symptoms and progress rapidly to life‐threatening complications such as intracerebral hemorrhage, maintaining a high index of clinical suspicion is essential. In this report, we describe a case of presumed CST complicated by extensive intracerebral hemorrhage in a young woman receiving long‐term OCP therapy, and we discuss the diagnostic challenge posed by the inability to perform advanced venous imaging because of the patient's clinical instability, along with the potential contribution of concurrent fasting‐related dehydration.

## 2 Case Presentation

### 2.1 Clinical Findings

A 34‐year‐old single woman was transferred to the emergency department of Hakim Hospital by emergency medical services due to decreased level of consciousness, recurrent seizures, and a one‐month history of pulsatile headache. She had been receiving low‐dose OCPs for approximately 1 year for the treatment of PCOS, including continued use while fasting during Ramadan; there was no history of cardiovascular or neurological disease, recent surgery, malignancy, venous thromboembolism, or family history of coagulation disorders. On the day of presentation, she developed a sudden severe headache with nausea and vomiting, unresponsive to analgesics, and was found unconscious approximately 3 h later.

On arrival, vital signs were: heart rate 100 beats/min, blood pressure 130/85 mmHg, respiratory rate 20 breaths/min, and oxygen saturation 88% on room air. She underwent endotracheal intubation because of hypoxemia and respiratory distress. Her Glasgow Coma Scale (GCS) score was E1V1M3 (total 5), and multiple seizure episodes were observed and treated with intravenous levetiracetam (500 mg), dexamethasone (16 mg), and mannitol (350 mL). Ocular examination revealed marked bilateral eyelid edema. The pupils were equal size but non‐reactive to light. Given the patient's critical condition and low GCS, a complete cranial nerve examination—including corneal and oculocephalic reflexes—could not be performed or documented, which limited the ability to characterize the neuro‐ophthalmological deficits in detail. ECG showed normal sinus rhythm. Laboratory findings are summarized in Table 1.

**TABLE 1:** Laboratory test results.

Lab test	Result	Unit	Normal range
WBC	8900	μL	4000–11,000
RBC	4.8	(×106/μL)	3.9–5.8
Hb	12	(g/dL)	12–17
HCT	37.3	(%)	32–48
Plt	183,000	μL	150,000–450,000
Neutrophil	91	(%)	
Lymphocyte	8	(%)	
Monocyte	1	(%)	
RDW	15.1	(%)	11.5–14.5
MCV	78.3	Fl	70–100
MCH	25.1	(pg)	31–36
MCHC	32.17	(g/dL)	32–36
PT patient	12	sec	11–14
PT			
INR	1		0.8–1.2
PTT	29	sec	21–37
BUN	5	(mg/dL)	7–20
Cr	0.8	(mg/dL)	0.6–1.3
Na	138	(mEq/L)	135–147
K	4.4	(mEq/L)	3.5–5
BS	86	(mg/dL)	70–135

### 2.2 Diagnostic Assessment

**FIGURE 1:**
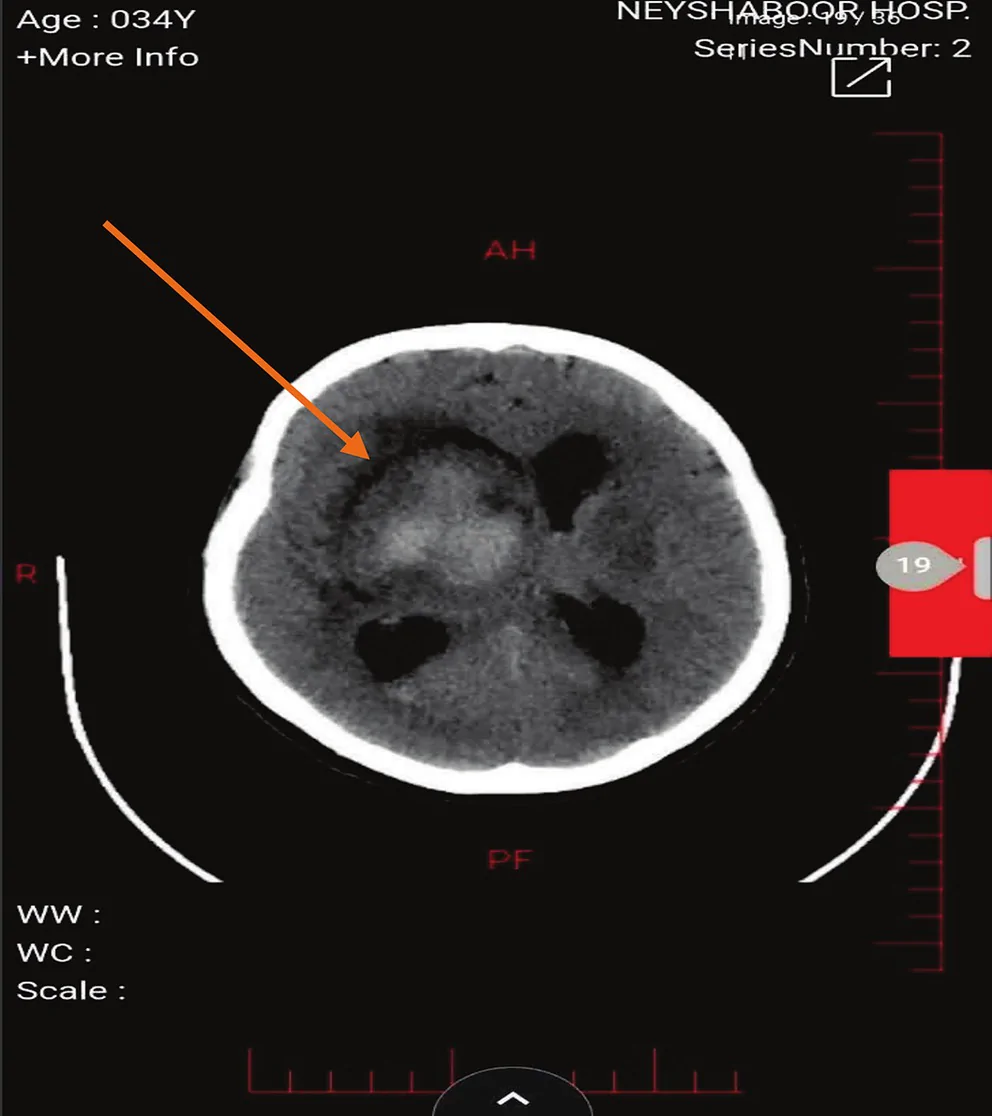
Non‐contrast CT scan of the head showed extensive ICH.

Non‐contrast brain CT demonstrated extensive intracerebral hemorrhage involving the brainstem with severe cerebral edema (Figure 1). Because of the patient's clinical instability, advanced venous imaging (CTV/MRV) could not be safely performed. Based on clinical presentation and CT findings, a presumptive diagnosis of unilateral cavernous sinus thrombosis (CST) was made.

### 2.3 Therapeutic Intervention and Outcome

**FIGURE 2:**
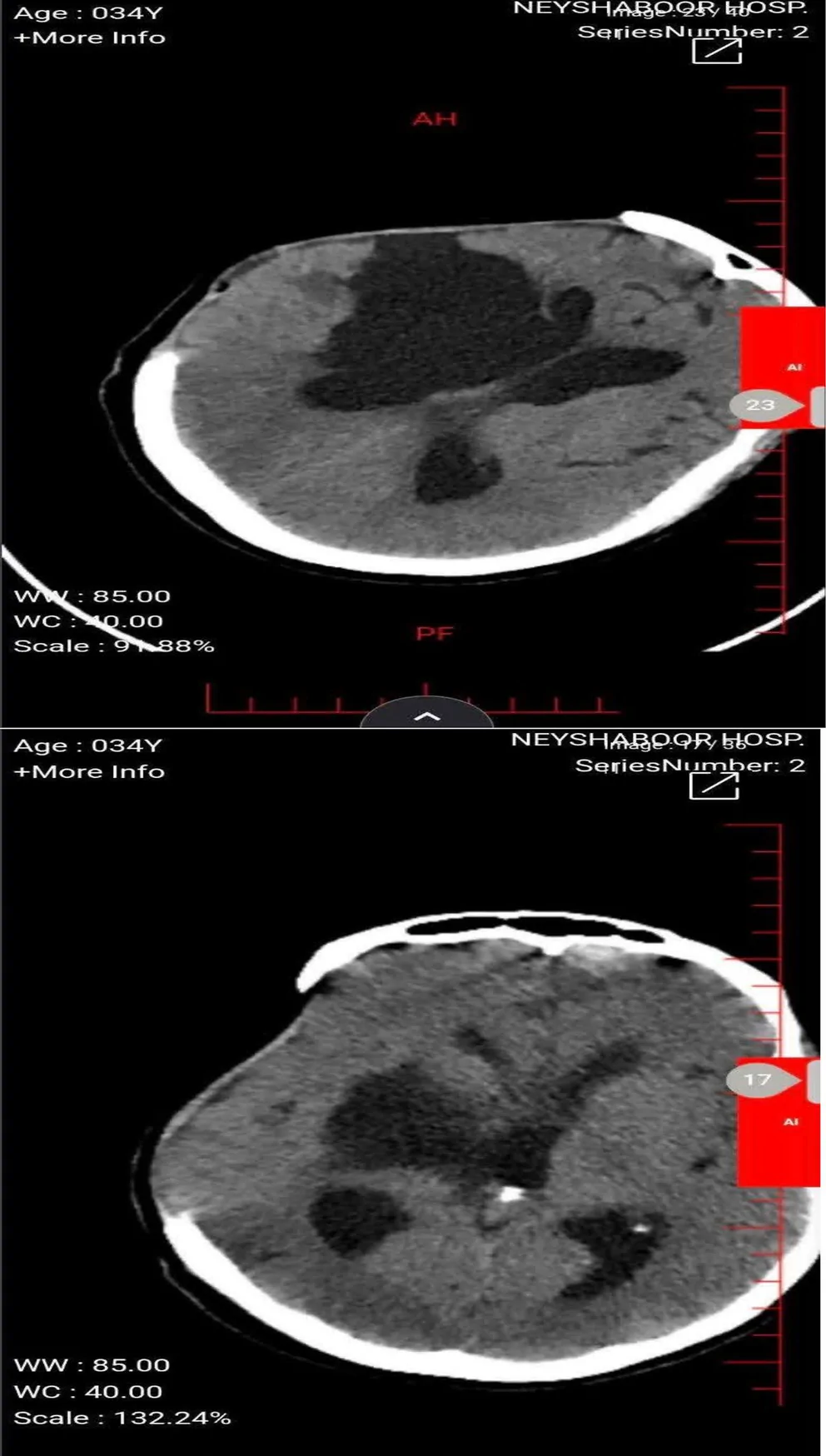
Post‐decompressive craniectomy and ICH evacuation.

Fallowing admission to the intensive care unit (ICU), her GCS deteriorated from 5 to 3. She received continuous intravenous unfractionated heparin monitored by aPTT, together with hypertonic saline and furosemide for cerebral edema control. Progressive neurological deterioration necessitated decompressive craniectomy with hematoma evacuation (Figure 2); coagulation parameters were reassessed preoperatively, and intraoperative ICP was managed with mannitol and end‐tidal CO2 monitoring.

**FIGURE 3:**
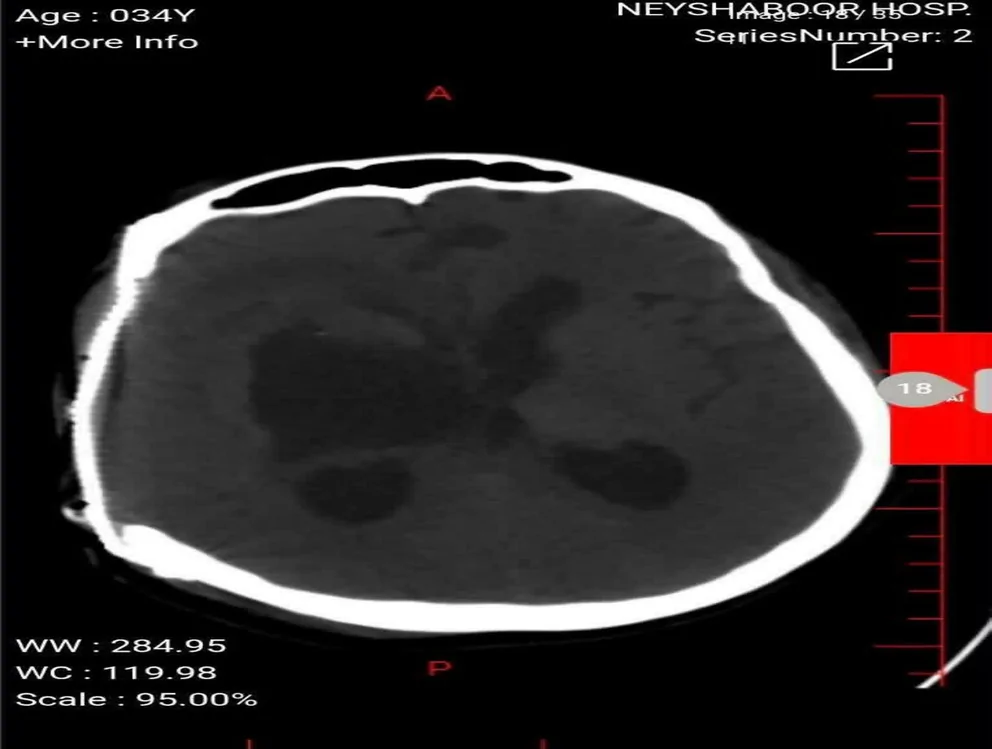
Post‐cranioplasty with titanium mesh.

**FIGURE 4:**
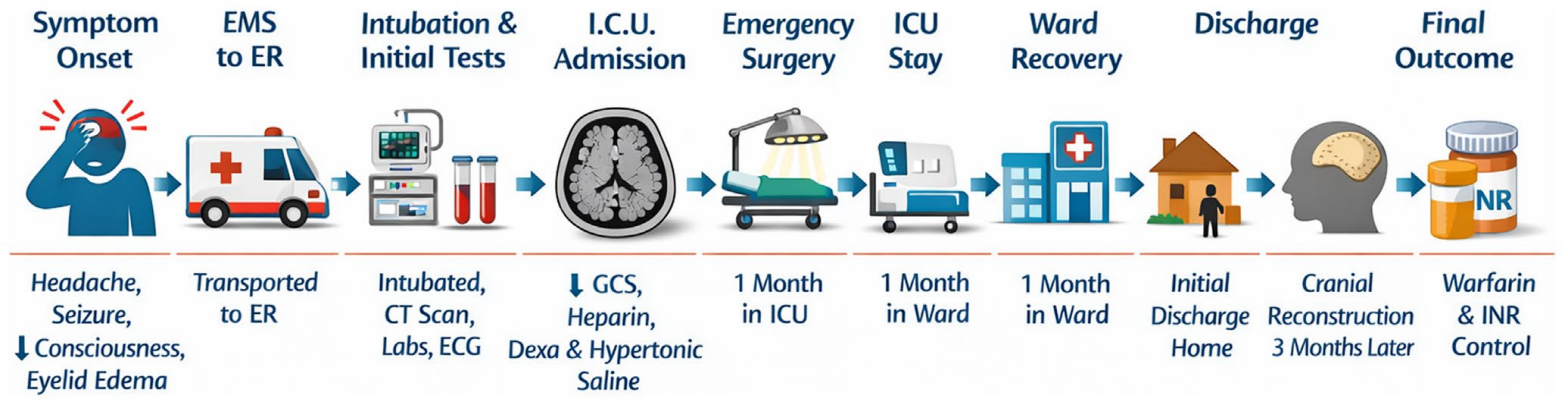
Clinical timeline from symptom onset to follow‐up.

By day 30, cerebral edema had decreased and GCS improved from 3 to 7. The patient was discharged after a total of 3 months of hospitalization with tracheostomy and nasogastric tubes and left‐sided hemiplegia; oral warfarin was initiated (target INR 2–3) for 3 months. One month after discharge, cranioplasty with titanium mesh was performed (Figure 3). With rehabilitation, the tracheostomy was removed, and oral feeding resumed. At the latest follow‐up, her GCS had improved to 15; however, residual neurological disability persisted (modified Rankin Scale score, 4). The overall clinical course is summarized in Figure 4.

## 3 Discussion

Cavernous sinus thrombosis (CST) can progress rapidly to life‐threatening complications if not promptly recognized, underscoring the importance of early clinical suspicion [10, 11]. In our patient, prolonged OCP use combined with concurrent fasting during Ramadan was identified as plausible contributing factors. Fasting‐related dehydration and hemoconcentration have previously been associated with an increased incidence of CVST, particularly among OCP users, and have been proposed to exacerbate the prothrombotic effects of OCPs on the coagulation system [12, 13, 14]; however, a direct causal relationship cannot be definitively established in this individual case. A broad differential diagnosis—including autoimmune disorders, inherited or acquired hypercoagulable states, malignancy, and infection—should be considered in any patient with suspected CVST. In our center, comprehensive thrombophilia and autoimmune screening could not be performed due to laboratory limitations, representing an additional diagnostic limitation in this case.

Clinically, CST typically presents with headache, periorbital edema, cranial nerve palsies, visual disturbances, seizures, and altered mental status, reflecting the close anatomical relationship between the cavernous sinus and cranial nerves III, IV, V, and VI [10]. Our patient's presentation—severe headache followed by seizures, reduced level of consciousness, and marked eyelid edema—was consistent with this pattern; however, the patient's critical neurological condition precluded a detailed cranial nerve and neuro‐ophthalmological examination, limiting the assessment of cranial nerve involvement associated with the suspected cavernous sinus thrombosis.

Neuroimaging remains the cornerstone of diagnosis of cerebral venous sinus thrombosis, including CST. Non‐contrast CT is typically the first modality used in emergency settings and may reveal indirect findings such as edema and hemorrhage, while CT venography, MRI/MRV, or catheter‐based cerebral angiography provide more definitive confirmation [2, 15, 16]. In our case, the patient's critical instability precluded advanced venous imaging, and the diagnosis of CST was therefore based on clinical presentation and supportive non‐contrast CT findings alone—an important limitation to consider when interpreting this case.

Management of CST generally follows the therapeutic principles established for CVST, focusing on the control of intracranial pressure, seizure management, and prevention of thrombus propagation. Anticoagulation with low‐molecular‐weight heparin (LMWH) or unfractionated heparin (UFH) remains the standard of care, even in the presence of intracerebral hemorrhage [2, 15]. Our patient was accordingly started on intravenous heparin with aPTT monitoring; however, progressive neurological deterioration necessitated decompressive craniectomy with hematoma evacuation, consistent with evidence that surgical decompression can be life‐saving in patients with severe intracranial hypertension refractory to medical therapy [2].

Prognosis in CVST depends largely on initial clinical severity, with coma at presentation and intracerebral hemorrhage recognized as poor prognostic factors [15, 17]. Despite these adverse features, our patient achieved substantial neurological recovery following combined medical and surgical management, suggesting that aggressive early intervention may improve outcomes even in severe presentations [11].

4

This case highlights the importance of considering cavernous sinus thrombosis as a possible complication in young women using OCPs, particularly in the setting of dehydration associated with fasting. A high index of clinical suspicion, supported by non‐contrast CT findings and the clinical context, facilitated early therapeutic decision‐making, including anticoagulation and, when indicated, surgical decompression. Despite an initially poor prognosis, timely multidisciplinary management resulted in meaningful functional recovery, thereby reinforcing the value of individualized, evidence‐based decision‐making in patients with suspected CST.

## Author Contributions

**Zohreh Akbari:** conceptualization, data curation, investigation, resources, software, visualization, writing – original draft. **Ali Salami:** conceptualization, data curation, methodology, resources, supervision, validation. **Ali Movahedi:** conceptualization, data curation, software, validation. **Seyyedeh Maryam Seyyedy:** conceptualization, data curation, methodology, resources, validation. **Kiana Babaei:** conceptualization, data curation, formal analysis, supervision, writing – original draft.

## Funding

The authors disclosed receipt of financial support for the research, authorship, and/or publication of this article from Neyshabur University of Medical Sciences, IR.NUMS.REC.1403.023.

## Ethics Statement

This study was approved by the Research Ethics Committee of Neyshabur University of Medical Sciences (decree code: IR.NUMS.REC.1403.023).

## Consent

The participant and her parents who were willing to take part in the study signed the informed consent form. Written informed consent was obtained from the patient for publication of this case report and any accompanying images.

## Conflicts of Interest

The authors declare no conflicts of interest.

## Data Availability

The authors declare that all the data supporting the findings of this study are available within the article.
